# Resting-State Neural-Activity Alterations in Subacute Aphasia after Stroke

**DOI:** 10.3390/brainsci12050678

**Published:** 2022-05-22

**Authors:** Xiaohui Xie, Ting Zhang, Tongjian Bai, Chen Chen, Gong-Jun Ji, Yanghua Tian, Jinying Yang, Kai Wang

**Affiliations:** 1 Department of Neurology, The First Affiliated Hospital of Anhui Medical University, Anhui Medical University, Hefei 230032, China; xiexiaohui0318@126.com (X.X.); zhangting9306@163.com (T.Z.); baiyunong1990@163.com (T.B.); 18225855669@163.com (C.C.); ayfytyh@126.com (Y.T.); 2 Anhui Province Key Laboratory of Cognition and Neuropsychiatric Disorders, Hefei 230032, China; jigongjun@163.com; 3 Collaborative Innovation Center of Neuropsychiatric Disorders and Mental Health, Hefei 230032, China; 4 The School of Mental Health and Psychological Sciences, Anhui Medical University, Hefei 230032, China; 5 Laboratory Center for Information Science, University of Science and Technology of China, Hefei 230026, China; jinying@ustc.edu.cn; 6 Institute of Artificial Intelligence, Hefei Comprehensive National Science Center, Hefei 231299, China

**Keywords:** stroke, subacute aphasia, ALFF, dynamic, FC

## Abstract

Linguistic deficits are frequent symptoms among stroke survivors. The neural mechanism of post-stroke aphasia (PSA) was incompletely understood. Recently, resting-state functional magnetic resonance imaging (rs-fMRI) was widely used among several neuropsychological disorders. However, previous rs-fMRI studies of PSA were limited to very small sample size and the absence of reproducibility with different neuroimaging indexes. The present study performed comparisons with static and dynamic amplitude of low-frequency fluctuations (ALFF) and functional connectivity (FC) based on modest sample size (40 PSA and 37 healthy controls). Compared with controls, PSA showed significantly increased static ALFF predominantly in the bilateral supplementary motor area (SMA) and right hippocampus-parahippocampus (R HIP-ParaHip) and decreased static ALFF in right cerebellum. The increased dynamic ALFF in SMA and decreased dynamic ALFF in right cerebellum were also found in PSA. The static and dynamic ALFF in right cerebellum was positively correlated with spontaneous speech. The FC between the SMA and R HIP-ParaHip was significantly stronger in patients than controls and positively correlated with ALFF in bilateral SMA. In addition, the FC between the R HIP-ParaHip and the right temporal was also enhanced in patients and negatively correlated with repetition, naming, and comprehension score. These findings revealed consistently abnormal intrinsic neural activity in SMA and cerebellum, which may underlie linguistic deficits in PSA.

## 1. Introduction

Aphasia is a disabling linguistic deficit manifested by impaired speech expression and comprehension caused by injury to the language-dominant hemisphere. Epidemiological studies have found that approximately one-third of stroke survivors suffer from aphasia [1] and that 40% still display significant speech impairment after one year [2]. Furthermore, some patients bear lifetime disability [3]. Patients with post-stroke aphasia (PSA) commonly exhibit poor social communication and low work competence, making it difficult to fully engage in society [4] and placing a substantial burden on individual, families and social services [3]. A comprehensive understanding of the neural mechanism of PSA is essential to guide the treatment for rehabilitation doctor.

Evidence from human stroke motor recovery models shows that the first 90 days after stroke are a critical ‘window of opportunity’ for recovery [5]. This time window also applies to recovery from aphasia [6]. Recent studies showed the better efficacy of speech and language therapy (SLT) on acute and subacute aphasia [7,8], while another report suggested that intensive SLT beginning during acute aphasia could not improve efficacy than routine SLT [9]. Similarly, contradictory results were found in the efficacy of transcranial direct current stimulation on subacute PSA [10]. These inconsistencies may be the consequence of unclear neural mechanism or greater inter-subject heterogeneity. Hence, it is very important to explore the activity of local and remote language-related brain regions in subacute aphasia. Fortunately, neuroimaging studies increase the current understanding of local and whole brain activity.

Functional magnetic resonance imaging (fMRI) represents an ideal tool and is widely applied to reveal the brain mechanisms of neurological diseases [11]. Several fMRI studies have found that the recovery of aphasia mainly depend on the preservation and functional status of language-related brain regions [12,13]. Richter et al. [14] reported that the structure of the right hemisphere, traditionally considered as the non-dominant hemisphere for language, can be integrated into the language network after severe stroke.

Resting-state fMRI(rs-fMRI [15]) uses blood oxygenation level-dependent signals to measure neural activity. Compared with task-based fMRI, it is easy to complete even for patients with severe aphasia. The amplitude of low-frequency fluctuations (ALFF [16]) is a frequently used method reflecting spontaneous activity for the analysis of rs-fMRI data [17]. Previous studies have found significantly increased ALFF in the contralesional cortices within two weeks after stroke [18] that correlate with behavioral measures of language function [19]. Dynamic ALFF (dALFF [20]) measurement is a recently developed imaging application that can reflect the temporal flexibility of resting-state brain networks. Dynamics can capture uncontrolled but recurring patterns in brain networks that cannot be detected by static analysis [21]. A combination of static and dynamic analysis can complement static changes in the pathological states. Reduced dALFF [20] has been reported in the left inferior frontal gyrus triangle of disrupted language networks in acute aphasia patients.

Language functions are implemented in widely distributed neural networks, as measured with resting-state fMRI. Disrupted corresponding network connectivity may underlie language deficits of aphasia [22]. Functional connectivity (FC [23]) is also a fairly direct and simple method to measure the level of temporal co-activation based on the computation of Pearson’s correlation coefficients between two anatomically separate brain regions. Previous research showed that the FC was abnormal in acute aphasia compared with health participants [18].

In this study, we combined static and dynamic ALFF to explore the dynamics of spontaneous brain activity in subacute aphasia. Then, we measured FC to assess the cooperation of remote region after stroke-induced disruption. Furthermore, we examined the correlations between neuroimaging alterations and language function scores. We hypothesized that there would be a functional abnormality of undamaged language-related brain regions. These aberrant areas may underlie linguistic deficits in subacute PSA.

## 2. Materials and Methods

### 2.1. Participants

**Figure 1:**
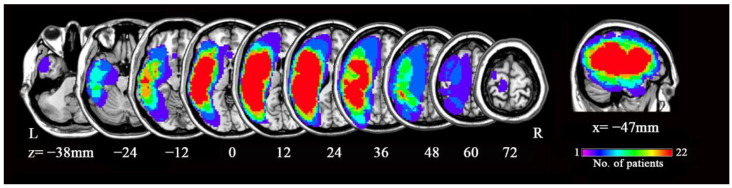
Regions of lesion overlap among subacute aphasia patients. Abbreviation: L, left; R, right; x and z refer to the x-plane and z-plane coordinates of the MNI space. Color bar indicates the number of patients.

We recruited 48 patients with post-stroke aphasia during subacute stage from the First Affiliated Hospital of Anhui Medical University and Anhui acupuncture hospital (Hefei, China) and 37 healthy controls (HCs) well matched for mean age, sex ratio, and years of education (see Table 1 for a summary of patient and HC demographics). Patients were recruited according to the following inclusion criteria: (i) 18–75 years old, (ii) first stroke in the left hemisphere (see Figure 1 for lesion overlap map), (iii) right-handed, (iv) native Chinese speaker, (v) aphasia persistent at day 1 after stroke, and (vi) post onset of stroke ≤3 months. Patients were excluded according to the following criteria: (i) any past or current neurological disease or family history of hereditary neurological disorders, (ii) mental illness or family history of hereditary mental disorders, (iii) severe dysarthria, (iv) a history of head injury or surgery, (v) alcohol or substance abuse, (vi) cerebral tumor or abscess, (vii) claustrophobia or implants incompatible with MRI, and (viii) failure to complete language testing or fMRI scans. In addition, excessive motion (>3 mm in translation or >3° in rotation) during scanning and abnormal brain structure were set as exclusion criteria for analysis but no data were excluded for these reasons. Eight patients were excluded for claustrophobia or incomplete language testing or fMRI. Finally, forty patients were enrolled in our study. HCs were recruited using the same exclusion criteria and inclusion criteria unrelated to stroke (i, iii, and iv).

### 2.2. Language Assessment

Before undergoing MRI scans, each participant received neurological examinations and neuropsychological assessments. Language deficit was assessed by the same professional language therapist using the Aphasia Battery of Chinese (ABC [24]), a Chinese standardized adaptation of the Western Aphasia Battery. The multidimensional test contains four subtypes, including spontaneous speech (semi-standardized interview, assessment of the fluency, and information of speech; total score 20), auditory comprehension (yes or no question, auditory picture matching task, and verbal instruction; total score 230), repetition (words and sentences; total score 100), and naming (simple objects, colors, pictures, and situations; total score 80). The combined score is used to calculate an aphasia quotient (AQ) reflecting the overall severity of language impairment. Patients with AQ below 93.8 [25] were considered as aphasic. The clinical characteristics of aphasia are summarized in Table 1.

The study conformed to the tenets of the Declaration of Helsinki and was approved by the Anhui Medical University Ethics Committee (2019H009). All patients and guardians were provided informed consent before enrolling.

### 2.3. Neuroimaging Data Acquisition

Neuroimaging data were acquired using an echo-planar imaging sequence on a 3.0-T MRI scanner (GE750w; GE Healthcare, Buckinghamshire, UK) at the University of Science and Technology of China, Anhui Province. During the MRI scan, participants were required to keep their heads still and eyes closed without falling asleep. All resting-state fMRI images were acquired using the following parameters: repetition time (TR) = 2400 ms, echo time (TE) = 30 ms, matrix size = 64 × 64, flip angle = 90°, field of view = 192 × 192 mm^2^, voxel size = 3 × 3 × 3 mm^3^, continuous slices = 46, and slice thickness = 3 mm. We also obtained a high-resolution 3D T1-weighted anatomical image using the following parameters: TR = 8.16 ms, TE = 3.18 ms, flip angle = 12°, field of view = 256 × 256 mm^2^, voxel size = 1 × 1 × 1 mm^3^, slice thickness = 1 mm, and 188 slices.

### 2.4. Lesion Mapping

We manually traced the outlines of lesions using MRIcron (http://www.mccauslandcenter.sc.edu/mricro/mricron, accessed on 20 March 2022) and then created a lesion mask for each patient in individual T1 image. After spatial normalization (described below), the superposition of all individual lesion masks was used to create a group-level lesion mask (see Figure 1). The group-level lesion mask was removed from the whole-brain mask as a segmentation mask using SPM8.

### 2.5. Neuroimaging Data Preprocessing

Functional images were preprocessed using the Data Processing Assistant for Resting-State fMRI (DPARSF: http://rfmri.org/DPARSF, accessed on 20 March 2022) [26] toolkit of the Statistical Parametric Mapping 8 (SPM8: http://www.fil.ion.ucl.ac.uk/spm, accessed on 20 March 2022) software package and the Resting State Functional MR Imaging Toolkit (REST: http://www.restfmri.net, accessed on 20 March 2022) [27]. We removed the first five functional volumes of data to ensure stable longitudinal magnetization, and corrected the remaining volumes for slice timing and head motion. Images with motion > 2.5 mm in translation or 2.5° in rotation were rejected. Individual 3D T1 images were co-registered to functional images. Next, we segmented 3D T1 images within the range of the segmentation mask. Structural T1 images were normalized to Montreal Neurological Institute (MNI) space based on the T1 image unified segmentation by 12-parameter nonlinear transformation. In addition, we used cost-function masking to discard the brain area of the group lesion, thereby avoiding divergence during spatial normalization [28]. This process must be conducted in SPM8 and has been used in other brain imaging studies including patients with lesions [29]. After spatial normalization, functional images were nuisance regressed with 24 Friston motion parameters, white matter high signals, cerebrospinal fluid signals, and global signals as regressors. Finally, images were spatially smoothed with a 4 mm full-width at half-maximum isotropic Gaussian kernel.

### 2.6. ALFF and dALFF Analysis

Amplitude of low-frequency fluctuation, a marker of resting-state intrinsic regional activity at the voxel level [30], was calculated using DPARSF. First, Fast Fourier Transform was used to convert the time series into a frequency power spectrum. For each voxel, we then obtained the average square root within the 0.01–0.1 Hz range. The whole-brain ALFF map of each individual was first calculated. Individual ALFF maps were standardized by Fisher’s z-score conversion to allow comparison between groups. We defined a specific group mask by subtracting the patient group level lesion mask from the whole-brain template. ALFF analysis was performed within the specific group mask in patients. Considering high heterogeneity of each participant, some of the patients had smaller lesions than the group level mask. Therefore, we re-defined another mask in which the voxels with less than 10 percent (4 patients) of patients were preserved to verify our results (see Supplementary Material).

Dynamic ALFF tracks the temporal changes in ALFF in the resting state using the Dynamic BC toolbox (www.restfmri.net/forum/DynamicBC, accessed on 20 March 2022) [31], which is based on the sliding-window analysis method. We segmented the full-length time series into a square sliding window of 20 TRs (48 s) as previous studies have shown that a window size of 30 to 60 s is a reasonable choice for capturing brain dynamics [32]. The step size of the sliding window was 1TR (2.4 s). This process produced 193 windows for each subject. In addition, we used two different window lengths (15 TRs [36 s] and 25 TRs [60 s]) to validate our results (see Figure S1 in Supplementary Material). The ALFF map obtained by all sliding windows was standardized using Fisher’s z-transformation to improve normality. Similarly, dALFF analysis was limited to the specific group mask in patients.

### 2.7. FC Analysis

**Figure 2:**
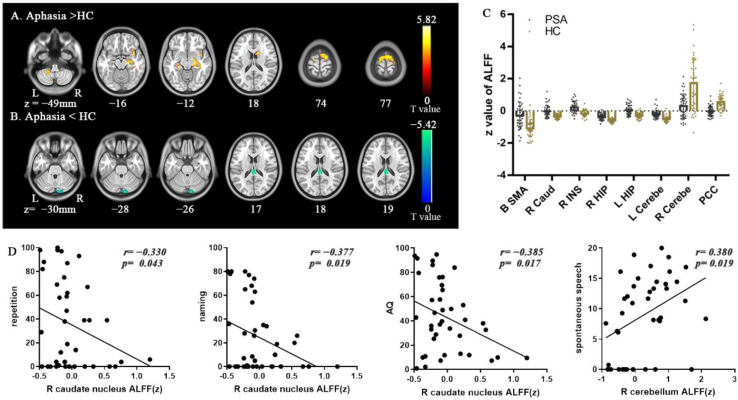
Brain regions displaying significant differences in static amplitude of low-frequency fluctuation (ALFF) between patients and healthy controls (HCs) and correlations between ALFF values and clinical scores in patients. All statistical maps were thresholded using a cluster-level family-wise error-corrected threshold of *p* < 0.05 (cluster-forming threshold at voxel-level *p* < 0.001). (**A**) Warm colors represent regions with increased ALFF values in aphasia. (**B**) Cold colors represent regions with decreased ALFF values in aphasia. Numbers below axial slices refer to the z-plane coordinates of the MNI space. Further details of these regions are shown in Table 2. (**C**) The z values of ALFF were extracted in significant regions between PSA and HC. (**D**) Static ALFF in the right caudate nucleus was negatively correlated with repetition, naming and AQ score; Static ALFF in the right cerebellum was positively correlated with spontaneous speech score (two-tailed, no correction). The *r* value denotes partial correlation coefficient. Abbreviations: PSA, post-stroke aphasia; Caud, caudate nucleus; INS, insula; Cerebe, cerebellum; PCC, bilateral posterior cingulate.

Functional connectivity was calculated using DPARSF, SPM8 and REST. According to the inter-group comparison of ALFF and dALFF, we defined the brain regions with significantly abnormal regions between two group as regions of interest (ROIs) for FC analyses (see Figure 2). For each participant, Pearson’s correlation coefficients were calculated between the mean time series of each ROI and the time series of every other brain voxel. To improve normality, correlation coefficients were transformed to z-values using Fisher’s r-to-z transformation, and FC maps were constructed for each individual.

### 2.8. Statistical Analysis

Two-sample t-tests were performed to identify the differences in standardized static ALFF and dALFF maps between patient and HC group using SPM8. The locations of the peak maxima within significant clusters were identified using REST. The ALFF and dALFF values in regions with differences were extracted. The same procedure was applied to compare FC between patients and HCs. All statistical maps were thresholded using a cluster-level family-wise error-corrected threshold of *p* < 0.05 (cluster-forming threshold at voxel-level *p* < 0.001). We performed correlation analysis to explore the relationship between aberrant ALFF and FC and clinical features. Considering high heterogeneity of each participant, the lesion volumes and time of post onset of stroke were regressed using partial correlation analysis. These analyses were performed in regions with statistically significant between-group difference.

## 3. Results

### 3.1. Demographic and Clinical Features

The demographic and clinical features of the patients and HCs are summarized in Table 1. There were no significant differences in mean age (t = 0.837, df = 75, *p* = 0.405), sex ratio (χ^2^ = 0.956, df = 1, *p* = 0.328), and mean educational level (t = −1.795, df = 75, *p* = 0.077) between the 40 aphasic patients and 37 HCs (Table 1). The overlap of brain lesions among patients is shown in Figure 1.

### 3.2. ALFF Contrast

**Figure 3:**
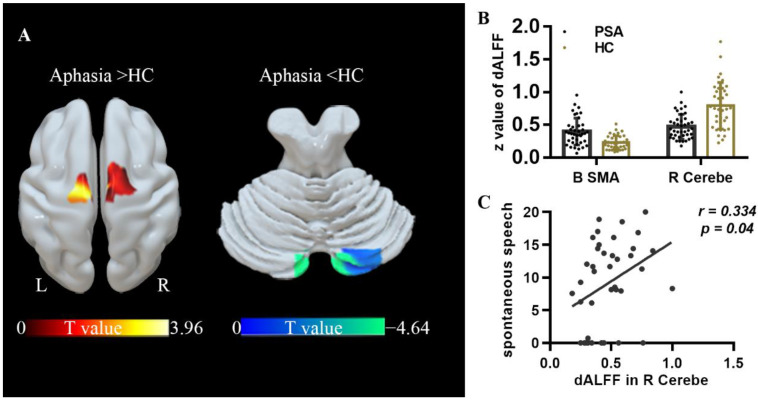
Brain regions displaying significant differences in dynamic ALFF (dALFF) between patients and healthy controls (HCs) and correlations between dALFF values and clinical scores in subacute aphasia patients. All statistical maps were thresholded using a cluster-level family-wise error-corrected threshold of *p* < 0.05 (cluster-forming threshold at voxel-level *p* < 0.001). (**A**) Warm and cold colors respectively represent regions with increased and decreased ALFF values in aphasia. Further details of these regions are shown in Table 3. (**B**) The z values of dALFF were extracted in significant regions between PSA and HC. (**C**) dALFF in the right cerebellum was positively correlated with spontaneous speech score (two-tailed, no correction). The *r* value denotes partial correlation coefficient.

Compared to HCs, patients displayed significantly greater ALFF in the R HIP-ParaHip, insula and caudate nucleus, left ParaHip-HIP, left cerebellum, and bilateral SMA, but significantly lower ALFF in the right cerebellum and bilateral posterior cingulate(PCC) (Table 2) (Figure 2). In the another mask, the results of ALFF analysis were similar (see Results in the Supplementary Material). Aphasia patients also exhibited significantly lower dALFF in the right cerebellum and greater dALFF in the B-SMA (Table 3) (Figure 3). Similarly, the results of other two different window lengths are shown in Figure S1 (see Results in the Supplementary Material).

### 3.3. FC Contrast

**Figure 4:**
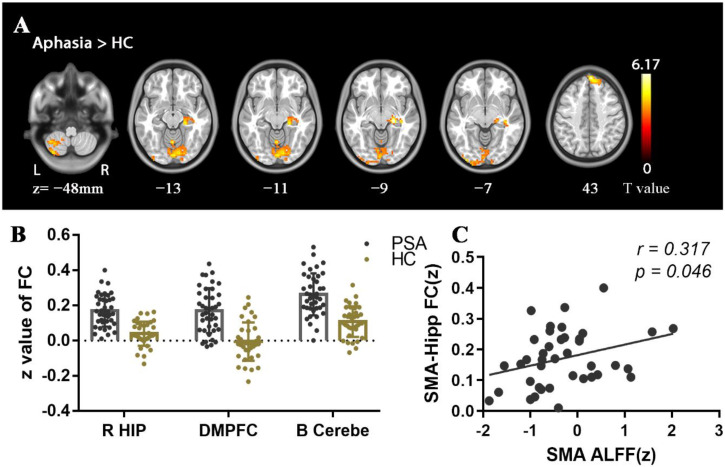
Abnormalities in SMA-based functional connectivity (FC) between PSA and HC and correlations between FC values and ALFF values of SMA in PSA. All statistical maps were thresholded using a cluster-level family-wise error-corrected threshold of *p* < 0.05 (cluster-forming threshold at voxel-level *p* < 0.001). (**A**) Warm colors represent regions with increased FC values in aphasia. Numbers below axial slices represent the z-plane coordinates of the MNI space. Further details of these regions are shown in Table 4. (**B**) The z values of FC were extracted in significant regions between PSA and HC. (**C**) ALFF in the SMA was positively corrected with FC between the SMA and R HIP-ParaHip (two-tailed, no correction).

**Figure 5:**
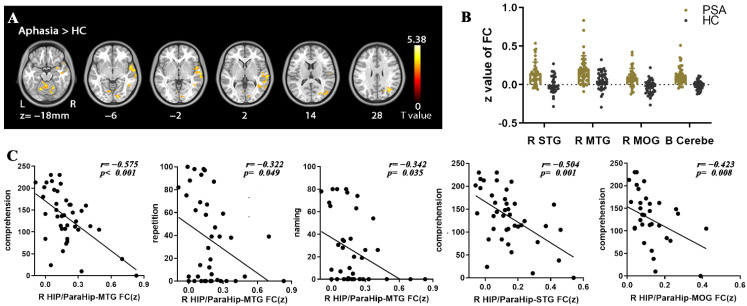
Abnormalities in R HIP/ParaHip-based functional connectivity (FC) between PSA and HC and correlations between FC values and clinical scores in PSA. All statistical maps were thresholded using a cluster-level family-wise error-corrected threshold of *p* < 0.05 (cluster-forming threshold at voxel-level *p* < 0.001). (**A**) Warm colors represent regions with increased FC values in aphasia. Numbers below axial slices represent the z-plane coordinates of the MNI space. Further details of these regions are shown in Table 4. (**B**) The z values of FC were extracted in significant regions between PSA and HC. (**C**) FC between the R HIP-ParaHip and right middle temporal gyrus (R MTG) was negatively correlated with comprehension, repetition, and naming score; FC between the R HIP-ParaHip and right superior temporal gyrus (R STG) and right middle occipital gyrus (R MOG) were negatively correlated with comprehension score in aphasia (two-tailed, no correction). The *r* value denotes partial correlation coefficient.

Function connectivity between the B SMA and R HIP-ParaHip, bilateral dorsomedial prefrontal cortex (DMPFC), and bilateral cerebellum were significantly stronger among aphasia patients compared with HCs (Table 4) (Figure 4). Patients also demonstrated stronger FC between the R HIP-ParaHip and right superior temporal gyrus (R STG), middle temporal gyrus (R MTG), middle occipital gyrus (R MOG), and bilateral cerebellum (Table 4) (Figure 5).

### 3.4. Correlational Analysis

In patients, static ALFF in the R caudate nucleus was negatively correlated with repetition, naming and AQ score (*r*= −0.330, *p* = 0.043; *r*= −0.377, *p* = 0.019; *r*= −0.385, *p* = 0.017) (Figure 2), while ALFF and dALFF in the right cerebellum were positively correlated with spontaneous speech score (*r* = 0.380, *p* = 0.019; *r* = 0.334, *p* = 0.04) (Figure 2 and Figure 3). In addition, FC between the R HIP-ParaHip and R MTG was negatively correlated with comprehension, repetition, and naming score (r= −0.575, *p* < 0.001; *r*= −0.322, *p* = 0.049; *r*= −0.342, *p* = 0.035). FC between the R HIP-ParaHip and the R STG and R MOG were negatively correlated with comprehension score (*r*= −0.504, *p* = 0.001; r = −0.423, *p* = 0.008) in aphasia (Figure 5). Finally, ALFF in the SMA was positively corrected with FC between the SMA and R HIP-ParaHip (*r* = 0.317, *p* = 0.046) (Figure 4).

## 4. Discussion

Linguistic ability depends on a distributed network of structures that is frequently disrupted by stroke, resulting in post-stroke aphasia. In the present study, we first applied two distinct neuroimaging indexes (static and dynamic ALFF) to explore regional neural alterations in subacute PSA. Our results suggested that PSA showed significantly increased static and dynamic ALFF in the bilateral SMA and decreased static and dynamic ALFF in right cerebellum. The consistent findings about static and dynamic ALFF in SMA and cerebellum may indicate crucial role in the linguistic loss or recovery of PSA.

Mounting evidence indicates that the SMA is involved in language processing [33], particularly of action language [34], and speech motor control [35]. Saur et al. reported stronger activation of the SMA in patients with subacute aphasia during auditory comprehension tasks compared to controls [36]. Due to limitations of our design, we are incapable of concluding the cognitive mechanism of the enhanced ALFF in SMA. The most likely explanation may be the maladaptive and diaschisis effect after large-regions lesion. Another slim odd is that this enhanced spontaneous activity in SMA played a compensatory role in subacute PSA. This deduction is based on an increasingly important network contribute to language recovery, i.e., domain-general networks [37,38], which includes SMA as key node. Of course, it is difficulty in understanding biological interpretations with brain susceptibility imaging, including rs-fMRI. It is also notable that the SMA is closely related with apraxia of speech [35,39], which frequently co-occur with aphasia among stroke survivors. However, the apraxia of speech was not assessed in present study, which may introduce confounding effect on our results. Future studies with more powerful tools are needed to probe the cognitive mechanism of local and connective alterations in SMA among PSA.

This study also revealed reduced ALFF/dALFF in the right cerebellum of aphasia patients. Traditional viewpoints focus the role of cerebellum on motor function. Recent evidence suggest the link between the cerebellum and a variety of linguistic processing, including language learning, semantic processing, and verbal memory [40]. Task-fMRI study in healthy subjects revealed that the activated cerebellar areas during language tasks present a right lateralizing, which is consistent with crossed projections of cerebro-cerebellar circuit [41,42]. The linguistic role of right cerebellum was also extensively studied in pathological conditions, including post-stroke aphasia. Li et al. [16] reported that the ALFF of right cerebellum was weaker in patients with aphasia compared to HCs. Another study found decreased ALFF in right cerebellum and weaker cerebrocerebellar connection [43]. Consistent with these findings, our results also replicated the decreased ALFF of right cerebellum in PSA. Moreover, using a relatively novel index (dALFF), we also found abnormity in the right cerebellum. The possible explanation is diaschisis effect [44] for this abnormal activity. Diaschisis refers to the neuronal dysfunction in undamaged regions distant to the lesion. We speculate that the decreased ALFF in right cerebellum may result from the poor interaction with cerebrum. Previous study found the relationship between the impaired language performance and the diaschisis effect [45] in the subacute aphasia. Similar diaschisis effect [38] has found in acute stage. Interestingly, recent studies also found that transcranial direct current stimulation to the right cerebellum improved language function [46]. These findings implied a potential therapeutic role of the right cerebellum in PSA.

Besides the abnormities in SMA and cerebellum found in present study, several temporal regions also present aberrant intrinsic activity in PSA, including increased ALFF in R HIP-ParaHip and increased FC between the R HIP-ParaHip and the R STG, R MTG and R MOG. Piai et al. reported that the hippocampus actively contributes to language comprehension [47]. Extensive studies have validated the critical role of the left temporal network [48,49] in language processing, including comprehension [50] and production [51] of speech. But temporal regions identified in present study mainly located in the right hemisphere. The role of the right hemisphere in aphasia has been debated for more than one century and remains controversial [52]. According to most studies supporting the “disinhibition hypothesis”, the increased activity may be related with severe lesion of left hemisphere [53,54]. However, a small group of studies also suggested the facilitative effect of the right hemisphere (right hemisphere hypothesis [55]). Based on this hypothesis, the enhanced connections within right temporal regions may reflect a compensation. Several factors confound the observation about the role of right hemisphere [38], including the location and extension of the left lesions, the time elapsed since the onset of aphasia. Studies with more homogeneous samples may be helpful to resolve this contradiction.

It is impossible to ignore the high heterogeneous sample in present study. Furthermore, additional limitations of our study should also be considered. First, a cross-sectional rather than longitudinal design limited the biological interpretations for our findings. Second, the lesion voxels for all patients were excluded from final analysis, which may lose sight of the alterations of perilesional tissue. Third, a relatively lenient statistical threshold was used in our study to test for the correlations between neural alterations and clinical variables. Correction for multiple correlations was not performed, which may potentially increase the risk of obtaining false-positive results. Finally, we did not assess the apraxia of speech, which may have an effect on results regarding the SMA.

## 5. Conclusions

Our results revealed the abnormal local activity in SMA and right cerebellum, as well as its dynamics. Furthermore, the increased connections were also found within temporal regions predominantly in the right. Although the biological interpretations remain unclear, these findings indicated a key role of SMA and right cerebellum in the language loss or recovery among PSA patients.

## Figures and Tables

**Table 1:** Demographic characteristics of patients and healthy controls.

	Patients	HCs	*p* Value
Sample size (n)	40	37	
Sex (male/female)	31/9	25/12	0.328 **
Age(years) ^§^	57.35 (11.79)	55.14 (11.39)	0.405 ^‡^
Education(years) ^§^	7.58 (5.09)	9.64 (4.97)	0.077 ^‡^
Handedness (left/right)	0/40	0/37	-
Lesion volume (cm^3^) ^††^	39.29 (14.69, 79.08)	-	-
Disease duration (weeks) ^††^	2(1, 5.75)	-	-
ABC scores		-	-
Spontaneous speech score ^††^	10.09 (0.19,14.35)	-	-
Auditory comprehension score ^§^	133.60 (59.96)	-	-
Repetition score ^††^	24 (0,68.50)	-	-
Naming score ^††^	9.25 (0,49.50)	-	-
AQ ^§^	43.21 (29.08)	-	-

Abbreviation: HCs, healthy controls. ABC, Aphasia Battery of Chinese. AQ, aphasia quotient. ** Chi-square test. ^‡^ Two-sample *t*-test. ^§^ Data are presented as the mean (standard deviation). ^††^ Data are presented as the median (interquartile range).

**Table 2:** Regions showing significant differences in static amplitude of low-frequency fluctuation (ALFF) between subacute aphasia patients and healthy controls (HCs).

	Brain Regions	MNI (x,y,z)	Cluster Size (Voxels)	T Value	*p* Value
Patients > HCs					
	R HIP-ParaHip	36, −48, 3	80	5.82	6.828 × 10^−8^
	B SMA	9, 0, 75	109	4.69	6.054 × 10^−6^
ALFF	L HIP-ParaHip	−27, −33, −9	54	5.66	1.324 × 10^−7^
	R insula	36, 6, −15	45	5.20	8.375 × 10^−7^
	R caudate nucleus	12, 9, 18	55	5.05	1.513 × 10^−6^
	L cerebellum	−15, −45, −45	219	5.53	2.235 × 10^−7^
Patients < HCs					
ALFF	R cerebellum	9 −90 −27	49	−5.01	1.741 × 10^−6^
	Bilateral posterior cingulate	0 −27 21	45	−5.42	3.537 × 10^−7^

Abbreviations: L, left; R, right; HIP-ParaHip, hippocampus-parahippocampus; SMA, supplementary motor area; MNI, Montreal Neurological Institute; x, y, z, coordinates of primary peak locations; T value, statistical value of peak voxel.

**Table 3:** Regions showing significant differences in dynamic amplitude of low-frequency fluctuation (dALFF) between subacute aphasia patients and healthy controls (HCs).

	Brain Regions	MNI (x,y,z)	Cluster Size (Voxels)	T Value	*p* Value
Patients > HCs					
dALFF	B SMA	21, 0, 75	45	3.96	8.495 × 10^−5^
Patients < HCs					
dALFF	R cerebellum	12, −90, −27	53	−4.64	7.315 × 10^−6^

Abbreviations: dALFF, dynamic amplitude of low-frequency fluctuation.

**Table 4:** Regions of interest (ROIs)-based functional connectivity (FC) abnormalities in subacute aphasia patients.

Seed Region	Connective Regions	MNI (x,y,z)	Cluster Size (Voxels)	T Value	*p* Value
Patients > HCs					
R HIP-ParaHip	R STG	63, −3, −9	270	4.67	6.484 × 10^−6^
	R MTG	54, −60 9	81	4.18	3.889 × 10^−5^
	R MOG	36, −63, 27	131	4.55	1.010 × 10^−5^
	Bilateral cerebellum	15, −42, −36	790	5.38	4.081 × 10^−7^
B SMA	R HIP-ParaHip	24, −24, −9	78	6.17	1.618 × 10^−8^
	DMPFC	3, 54, 42	140	5.74	9.441 × 10^−8^
	Bilateral cerebellum	−39, −60, −45	1620	5.54	2.139 × 10^−7^

Abbreviations: STG, superior temporal gyrus; MTG, middle temporal gyrus; MOG, middle occipital gyrus; DMPFC, dorsomedial prefrontal cortex.

## Data Availability

The data that support the findings of this study are available from the corresponding author upon reasonable request.

## Supplementary Materials

The following supporting information can be downloaded at: https://www.mdpi.com/article/10.3390/brainsci12050678/s1, Figure S1: Brain regions displaying significant differences in dALFF between patients and healthy controls; Figure S2: Brain regions displaying significant differences in static amplitude of low-frequency fluctuation (ALFF) between patients and healthy controls (HCs) and correlations between ALFF values and clinical scores in subacute aphasia patients; Figure S3: Brain regions displaying significant differences in dynamic ALFF (dALFF) between patients and HCs and correlations between dALFF values and clinical scores in subacute aphasia patients; Figure S4: Abnormalities in SMA-based FC between PSA and HC and correlations between FC values and ALFF values of SMA in PSA. Brain regions demonstrating FC differences between PSA and HC; Figure S5: Abnormalities in R HIP-based functional connectivity (FC) between PSA and HC. Brain regions demonstrating FC differences between PSA and HC; Table S1: Regions showing significant differences in static ALFF between patients and HCs; Table S2: Regions showing significant differences in dALFF between patients and HCs; Table S3: SMA-based FC abnormalities in subacute aphasia patients; Table S4: R HIP-based FC abnormalities in subacute aphasia patients.
