# Supplementary material for: Resting-State Neural-Activity Alterations in Subacute Aphasia after Stroke
Source: Brain Sci. 2022 May 22;12(5):678. doi: 10.3390/brainsci12050678 (PMC9139890; doi:10.3390/brainsci12050678)
Supplement: Supplementary file 1 [file brainsci-12-00678-s001.zip › brainsci-1667298-supplementary.pdf]

Supplementary Materials

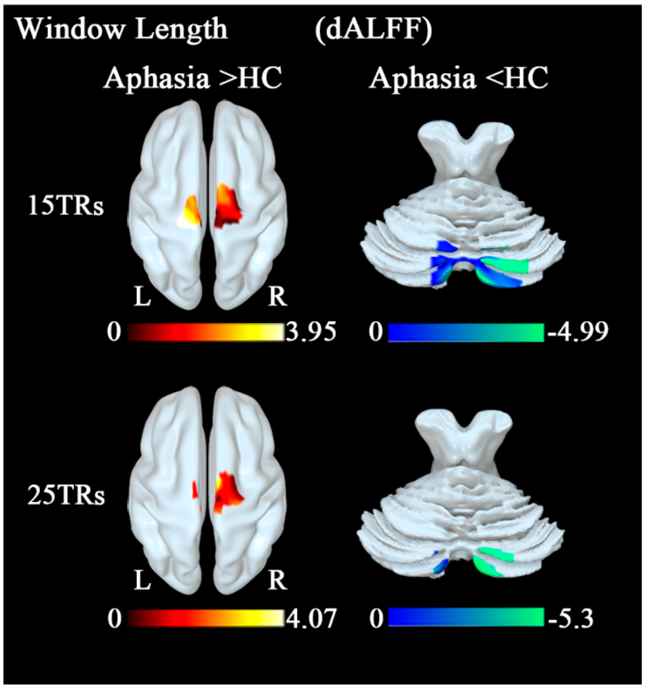

Figure S1. Brain regions displaying significant differences in dALFF between patients and healthy controls. All comparisons were performed using independent samples t-test within the specific group mask by subtracting the patient group level lesion mask from the whole-brain template. All statistical maps were thresholded using a cluster-level family-wise error-corrected threshold of $p < 0.05$ (cluster-forming threshold at voxel-level $p < 0.001$). Warm/Cold colors represent regions with increased/decreased dALFF values in aphasia, respectively. The step size of the sliding window is 1 repetition time (TR) (2.4 s). The 15/25 TRs produced 198 /188 windows for each subject.

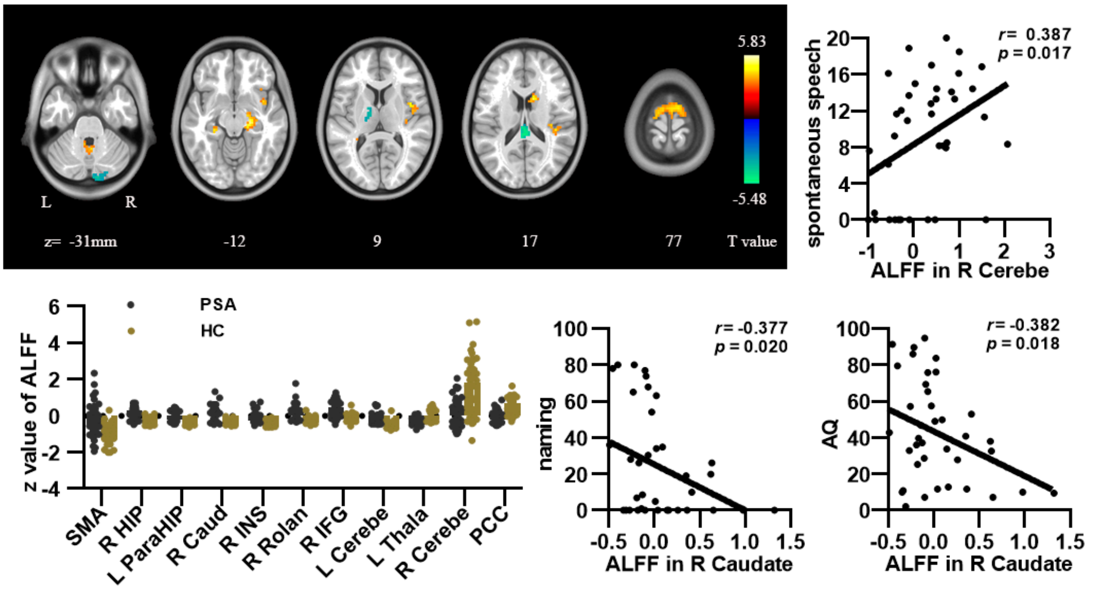

Figure S2. Brain regions displaying significant differences in static amplitude of low-frequency fluctuation (ALFF) between patients and healthy controls (HCs) and correlations between ALFF values and clinical scores in subacute aphasia patients. All comparisons were performed using independent samples t-test within another mask that the voxels with less than 10 percent (4 patients) of patients were preserved. All statistical maps were thresholded using a cluster-level family-wise error-corrected threshold of $p < 0.05$ (cluster-forming threshold at voxel-level $p < 0.001$). Warm/ Cold colors represent regions with increased/ decreased ALFF values in aphasia, respectively. Numbers below axial slices refer to the z-plane coordinates of the MNI space. Further details of these regions are shown in Table S1. The z values of ALFF were extracted in significant regions between PSA and HC. Static ALFF in the right caudate nucleus was negatively correlated with naming and AQ score; Static ALFF in the right cerebellum was positively correlated with spontaneous speech score(two-tailed, no correction). The r value denotes partial correlation coefficient.

Abbreviations: PSA, post-stroke aphasia; SMA, supplementary motor area, HIP, hippocampus, Caud, caudate nucleus; INS, insula; Rolan, Rolandic, IFG, Inferior Frontal Gyrus, Cerebe, cerebellum; Thala, Thalamus, PCC, posterior cingulate.

Table S1. Regions showing significant differences in static ALFF between patients and HCs

	Brain regions	MNI(x,y,z)	Cluster size(voxels)	T value	*p* value
		Patients >	HCs		
ALFF	SMA	9,0,75	94	4.554	$9.975*10^{-6}$
	R HIP	21,-21,-15	54	5.834	$6.447*10^{-8}$
	L ParaHIP	-27,-33,-9	67	5.220	$7.717*10^{-7}$
	R caudate nucleus	12,9,18	38	4.898	$2.714*10^{-6}$
	R INS	33,-24,15	27	4.662	$6.666*10^{-6}$
	R Rolandic_oper	48,-6,6	36	4.505	$1.197*10^{-5}$
	R IFG	36,6,-15	38	4.941	$2.295*10^{-6}$
	L cerebellum	-15,-45,-45	104	5.194	$8.560*10^{-7}$
		Patients < HCs			

	L Thalamus	-12,-9,12	29	-4.217	$3.417*10^{-5}$
ALFF	R cerebellum	9,-90,-27	49	-4.944	$2.272*10^{-6}$
	PCC	-3,-27,21	48	-5.482	$2.714*10^{-7}$

Abbreviations: L, left; R, right; MNI, Montreal Neurological Institute; x, y, z, coordinates of primary peak locations; T value, statistical value of peak voxel.

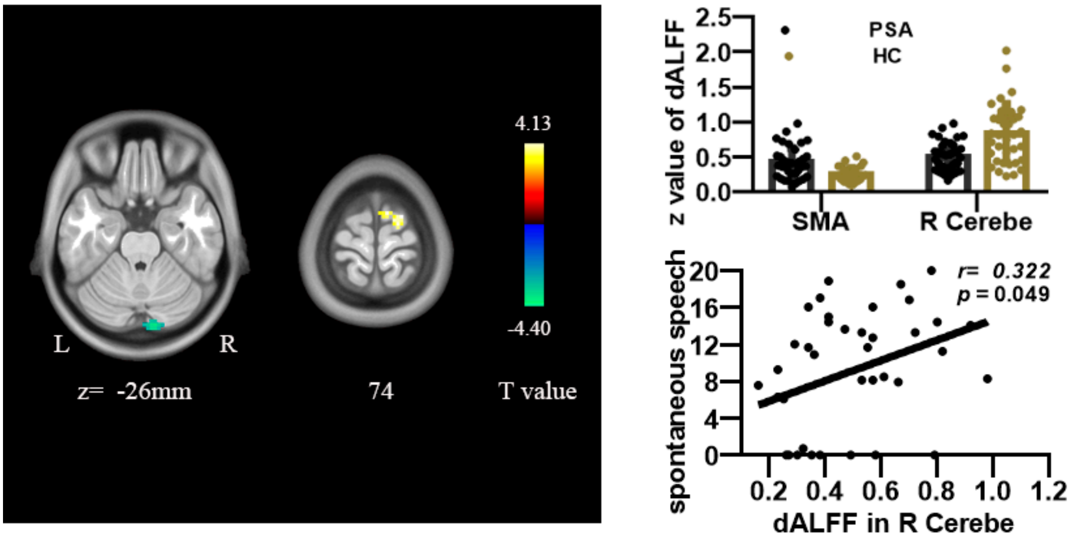

Figure S3. Brain regions displaying significant differences in dynamic ALFF (dALFF) between patients and HCs and correlations between dALFF values and clinical scores in subacute aphasia patients. All comparisons were performed using independent samples t-test within another mask that the voxels with less than 10 percent (4 patients) of patients were preserved. All statistical maps were thresholded using a cluster-level family-wise error-corrected threshold of $p < 0.05$ (cluster-forming threshold at voxel-level $p < 0.001$). Warm/ Cold colors represent regions with increased/ decreased dALFF values in aphasia, respectively. Numbers below axial slices refer to the z-plane coordinates of the MNI space. Further details of these regions are shown in Table S2. The z values of dALFF were extracted in significant regions between PSA and HC. dALFF in the right cerebellum was positively correlated with spontaneous speech score (two-tailed, no correction). The r value denotes partial correlation coefficient.

Table S2. Regions showing significant differences in dALFF between patients and HCs

	Brain regions	MNI(x,y,z)	Cluster size(voxels)	T value	*p* value
dALFF	Patients > HCs				
	SMA	21,0,75	52	4.132	$4.629*10^{-5}$
	Patients < HCs				
	R cerebellum	12,-90,-27	36	-4.400	$1.765*10^{-5}$

Abbreviations: dALFF, dynamic amplitude of low-frequency fluctuation.

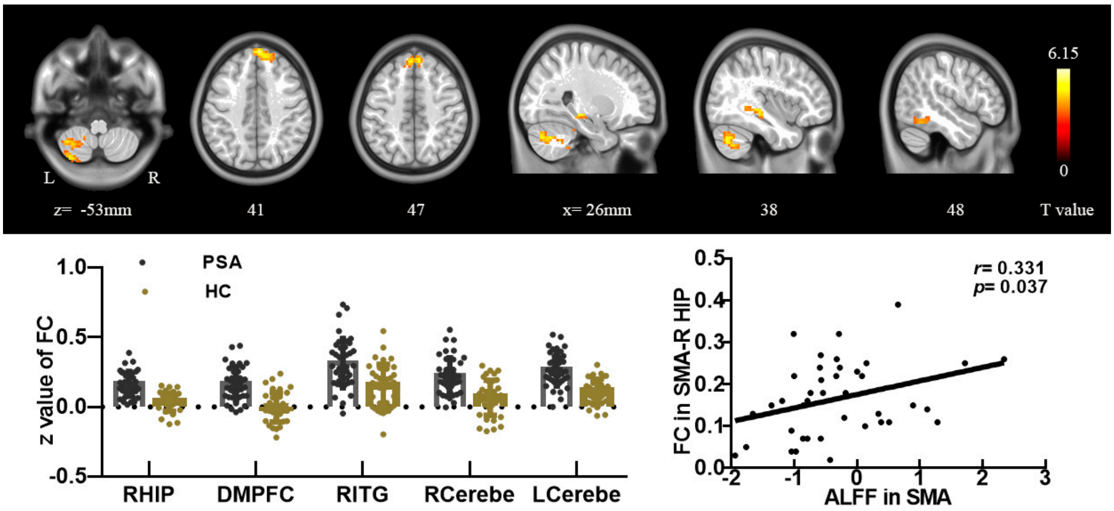

Figure S4. Abnormalities in SMA-based FC between PSA and HC and correlations between FC values and ALFF values of SMA in PSA. Brain regions demonstrating FC differences between PSA and HC. All comparisons were performed using independent samples t-test within another mask that the voxels with less than 10 percent (4 patients) of patients were preserved. All statistical maps were thresholded using a cluster-level family-wise error-corrected threshold of $p < 0.05$ (cluster-forming threshold at voxel-level $p < 0.001$). Warm colors represent regions with increased FC values in aphasia. Numbers below axial and sagittal slices represent the z-plane and x-plane coordinates of the MNI space. Further details of these regions are shown in Table S3. The z values of FC were extracted in significant regions between PSA and HC. ALFF in the SMA was positively corrected with FC between the SMA and R HIP (two-tailed, no correction). Abbreviations: DMPFC, dorsomedial prefrontal cortex. ITG, Inferior Temporal Gyrus.

Table S3. SMA-based FC abnormalities in subacute aphasia patients

	Brain regions	MNI(x,y,z)	Cluster size(voxels)	T value	*p* value
		Patients > HCs			
FC	R HIP	24,-24,-9	81	6.146	$1.763*10^{-8}$
	DMPFC	3,54,42	162	5.506	$2.462*10^{-7}$
	R ITG	54,-60,-15	40	4.109	$5.025*10^{-5}$
	R cerebellum	36,-69,-39	216	5.350	$4.609*10^{-7}$
	L cerebellum	-36,-63,-45	1417	5.549	$2.067*10^{-7}$

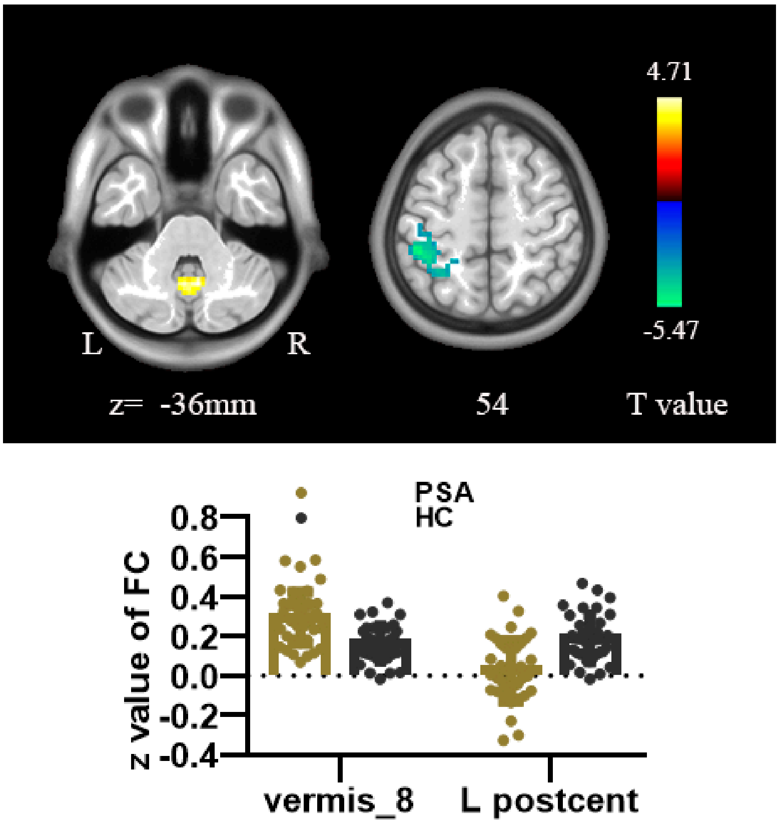

Figure S5. Abnormalities in R HIP-based functional connectivity (FC) between PSA and HC. Brain regions demonstrating FC differences between PSA and HC. All comparisons were performed using independent samples t-test within another mask that the voxels with less than 10 percent (4 patients) of patients were preserved. All statistical maps were thresholded using a cluster-level family-wise error-corrected threshold of $p < 0.05$ (cluster-forming threshold at voxel-level $p < 0.001$). Warm/ Cold colors represent regions with increased/ decreased FC values in aphasia, respectively. Numbers below axial slices represent the z-plane coordinates of the MNI space. Further details of these regions are shown in Table S4. The z values of FC were extracted in significant regions between PSA and HC. Abbreviations: postcent, postcentral.

Table S4. R HIP-based FC abnormalities in subacute aphasia patients

	Brain regions	MNI(x,y,z)	Cluster size(voxels)	T value	*p* value
		Patients > HCs			
FC	Vermis_8	6,-60,-36	42	4.708	$5.596*10^{-6}$
		Patients < HCs			
	L_postcentral	-45,-39,54	135	-5.474	$2.803*10^{-7}$
